# Integrative analysis and identification of key elements and pathways regulated by Traditional Chinese Medicine (Yiqi Sanjie formula) in colorectal cancer

**DOI:** 10.3389/fphar.2022.1090599

**Published:** 2022-12-13

**Authors:** Xianghui Wan, Fangfang Tou, Jiquan Zeng, Xinyi Chen, Shanshan Li, Lanyu Chen, Zhi Zheng, Jun Rao

**Affiliations:** ^1^ Jiangxi Cancer Hospital, The Second Affiliated Hospital of Nanchang Medical College, Nanchang, Jiangxi, China; ^2^ Jiangxi Provincial People’s Hospital, The First Affiliated Hospital of Nanchang Medical College, Nanchang, Jiangxi, China; ^3^ Department of Hematology and Oncology, Beijing University of Chinese Medicine, Beijing, China

**Keywords:** colorectal cancer, Yiqi Sanjie formula, peiminine, gut microbiota, inflammation

## Abstract

**Introduction:** The clinical efficacy of Yiqi Sanjie (YQSJ) formula in the treatment of stage III colorectal cancer (CRC) has been demonstrated. However, the underlying antitumor mechanisms remain poorly understood.

**Materials and methods:** The aim of the present study was to comprehensively characterize the molecular and microbiota changes in colon tissues and fecal samples from CRC mice and in CRC cell lines treated with YQSJ or its main active component, peiminine. Integrative tandem mass tag-based proteomics and ultra-performance liquid chromatography coupled with time-of-flight tandem mass spectrometry metabolomics were used to analyze azoxymethane/dextran sulfate sodium-induced CRC mouse colon tissues.

**Results:** The results showed that 0.8% (57/7568) of all detected tissue proteins and 3.2% (37/1141) of all detected tissue metabolites were significantly changed by YQSJ treatment, with enrichment in ten and six pathways associated with colon proteins and metabolites, respectively. The enriched pathways were related to inflammation, sphingolipid metabolism, and cholesterol metabolism. Metabolomics analysis of fecal samples from YQSJ-treated mice identified 121 altered fecal metabolites and seven enriched pathways including protein digestion and absorption pathway. 16S rRNA sequencing analysis of fecal samples indicated that YQSJ restored the CRC mouse microbiota structure by increasing the levels of beneficial bacteria such as Ruminococcus_1 and Prevotellaceae_UCG_001. In HCT-116 cells treated with peiminine, data-independent acquisition-based proteomics analysis showed that 1073 of the 7152 identified proteins were significantly altered and involved in 33 pathways including DNA damage repair, ferroptosis, and TGF-β signaling.

**Conclusion:** The present study identified key regulatory elements (proteins/metabolites/bacteria) and pathways involved in the antitumor mechanisms of YQSJ, suggesting new potential therapeutic targets in CRC.

## Introduction

Colorectal cancer (CRC) accounts for approximately 10% of all cancers and remains the second leading cause of cancer-related death worldwide (Ciardiello et al., 2022). The global incidence of CRC has increased in recent years, which poses a severe public health challenge. In 2020, an estimated 1.9 million new CRC cases and 0.9 million CRC-related deaths were reported worldwide. Most patients with stage I–III CRC are treated with radical resection (Catalano et al., 2022). Recurrence and metastasis of CRC are major causes of CRC-related death. Hence, post-surgery adjuvant radiotherapy and chemotherapy are routinely administered to these patients. Patients with metastatic stage IV CRC, which is unsuitable for radical resection, have several systemic treatment options, including chemotherapy regimens (oxaliplatin, 5-fluorouracil, and irinotecan) and targeted agents (bevacizumab, aflibercept, cetuximab, and panitumumab) (El Bali et al., 2021). However, surgery, radiotherapy, and chemotherapy treatments can impair immune functions or induce adverse reactions or multidrug resistance, thus negatively affecting quality of life. Therefore, the identification of effective chemotherapeutic options with fewer side effects remains an urgent need for CRC patients.

Traditional Chinese Medicine (TCM) is widely used in East Asia for the supplementary treatment of various cancers, including non-small cell lung cancer (NSCLC), triple-negative breast cancer (TNBC), pancreatic cancer, and CRC (Li et al., 2021a; Gao et al., 2021; Yang et al., 2021). Increasing evidence indicates that TCM can increase the efficacy of chemotherapy by reducing side effects and toxicity. For example, supplementary treatment with TCM after standard therapy in NSCLC relieves clinical symptoms and improves the quality of life of CRC patients (Li et al., 2021a; 2021b). The most common theory explaining the activity of TCM is that in the presence of a weakened immune system and strong tumor growth, TCM can help reverse the imbalance by “strengthening the body” and “eliminating evil.” Many classical TCM preparations, including Sijunzi decoction, Huangqi Jianzhong tang, Xiao Tan He Wei decoction, and Weipixiao decoction, have shown efficacy in the treatment of precancerous lesions of gastric cancer (PLGC) in clinical practice. In the treatment of PLGC, TCM acts mainly by regulating cell proliferation and apoptosis (Xu et al., 2022). The role of TCM in the treatment of CRC has been reported, and data support the benefits of TCM herbals as a long-term therapeutic strategy for patients with stage II and III CRC (Li-Weber, 2013; Li et al., 2021b; Tang et al., 2021). TCM can interact with gut microbiota, which has a considerable influence on the therapeutic effects (Feng et al., 2021). For example, the classic TCM sini decoction ameliorates CRC symptoms and modulates the intestinal microbiota composition of CRC mice by regulating intestinal immune responses and protecting the colonic mucosal barrier.

“Omics” technologies, including transcriptomics, proteomics, and metabolomics, have widely contributed to elucidating the therapeutic mechanisms underlying different diseases including coronavirus disease (COVID-19) and cancers (Ma et al., 2022; Visal et al., 2022). Proteins are the fundamental constituents of all cells, and proteomics studies aim to identify and quantify all proteins in given biological samples. Xu et al. (2020) elucidated the underlying mechanism of Xihuang pill (XHP, a traditional Chinese herbal formula) against TNBC using a combination of pharmacology and proteomics methods. Similar to proteomics, metabolomics is another powerful omics approach for the detection of small molecule metabolites in given biological samples. It has been widely used for identifying the molecular targets of TCM and developing new therapeutic agents (Luo et al., 2020). Integrative network pharmacology and metabolomics analyses were used to identify tricin as the active anticancer component of Weijing decoction, which can suppress lung cancer growth by inhibiting protein kinase C alpha (PRKCA) and sphingolipid signaling (Li et al., 2021). Integrative multi-omics analyses are more effective than a single “-omic” study for understanding the molecular characteristics of diseases and therapeutic responses. Zhang et al. (2017) used high-throughput metabolomics and proteomics methods to determine the metabolite and protein changes in the kidney-yang deficiency syndrome rat model; they identified the mechanisms underlying the effect of the Jinkui Shenqi pill, and showed that it affects the Wnt, PPAR, chemokine, and MAPK signaling pathways.

We previously demonstrated that together with chemical and/or targeted drugs, the Yiqi Sanjie (YQSJ) formula is clinically effective in the treatment of stage III CRC (Zhang et al., 2015; Zheng et al., 2022). The addition of YQSJ treatment to mFOLFOX6 postoperative adjuvant chemotherapy significantly reduces side effects such as gastro-intestinal reactions, hematologic toxicity, and peripheral neurotoxicity. YQSJ contributed to prolonging the survival of CRC patients with a high 3-year disease free survival (DFS) rate. Integrative proteomics and metabolomics analyses were performed on serum samples collected from 20 advanced CRC patients who were treated with the combination of traditional Chinese and Western medicine. YQSJ decreased the levels of serum IGF-1 and prevented the loss of Mg and liver damage resulting from chemical and/or targeted drugs including cetuximab and FOLFOX6. In the present study, we used integrated proteomics and metabolomics methods to characterize the mechanisms underlying the therapeutic effects of YQSJ and its main active component peiminine on colon tissues of azoxymethane (AOM)/dextran sulfate sodium (DSS)-induced CRC mice treated with YQSJ. In addition, a data-independent acquisition (DIA)-based-proteomics approach was used to determine the protein changes in HCT-116 colon cancer cells treated with peiminine. The -omics results were integrated with those of 16S rRNA sequencing analysis of mouse fecal samples to explore the role of gut microbes in the treatment effects mediated by the YQSJ formula.

## Materials and methods

### Animal experiments

Male Balb/c mice (6–8 weeks of age) were bred in a specific pathogen-free (SPF) facility. After acclimating for 1 week, the mice were randomly divided into three groups with three mice in each group: control (CTL) group, CRC model (CRC) group, and YQSJ + CRC model (YQSJ) group. The animal experimental protocol was similar to the one that described previously (Wang et al., 2021). In the YQSJ group, each mouse was administered 0.4 ml YQSJ orally (1.5 g/ml) each day from day 28 to day 67. As reported previously, the YQSJ formula contained *Fritillaria thunbergii Miq*. [Liliaceae], *Codonopsis pilosula* (*Franch*). *Nannf.* [Campanulaceae], *Atractylodes macrocephala Koidz.* [Asteraceae], *Coix lacryma-jobi* var. *Ma-yuen* (*Rom.Caill*). *Stapf* [Poaceae], *Wisteriopsis reticulata* (*Benth*). *J.Compton and Schrire* [Fabaceae], *Polygonatum sibiricum Redouté* [Asparagaceae], *Angelica sinensis* (*Oliv*). *Diels* [Apiaceae], *Lycium chinense Mill.* [Solanaceae], and *Wurfbainia villosa* (*Lour*). *Skornick*. and *A.D*. *Poulsen* [Zingiberaceae] (Zhang et al., 2015; Zheng et al., 2022). The monarch drug in the formula was *Fritillaria thunbergii* (Chinese name: Zhebeimu) and its main active component was peiminine (37.30 min), which was determined *via* liquid chromatography (LC) analysis (Zheng et al., 2017). The results were verified by the Traditional Chinese Medicine Systems Pharmacology Database and Analysis Platform (TCMSP, http://sm.nwsuaf.edu.cn/lsp/tcmsp.php). The chemical composition of YQSJ complies with the ConPhyMP statement and has been validated for classification at “http://mpns.kew.org/mpns-portal” (Heinrich et al., 2022). Mice in the CTL and CRC groups were given equal volumes of physiological saline during the same period. The body weight of each mouse was recorded every day. After sacrifice on day 67, the colon tissues of each mouse were collected. Mouse fecal samples were collected before treatment on days 27–28 and after treatment on days 66–67 (the endpoint).

### Integrative proteomics and metabolomics analyses of colon tissues

Integrative proteomics and metabolomics analyses of colon tissues were performed by the Applied Protein Technology Co. (Shanghai, China) (Ni et al., 2021). In the tandem mass tag (TMT)-based proteomics analysis, proteins were extracted in sodium dodecyl sulfate (SDS)-DL-dithiothreitol (DTT) buffer containing 100 mM Tris-HCl (pH 7.6), 1 mM DTT, and 4% SDS. The amount of protein was quantified using the BCA protein assay kit (Bio-Rad, United States). Protein digestion was performed using filter-aided sample preparation (FASP). The TMT reagent was applied to label 100 μg of peptide mixtures from each sample according to the manufacturer’s instructions. The labeled peptides were mixed, fractionated by strong cation exchange (SCX) chromatography, and subjected to LC-MS/MS analysis using the EASY-nLC 1000 Liquid Chromatograph (Thermo Fisher Scientific, Waltham, MA, United States) connected to a Q-Exactive Mass Spectrometer (Thermo Fisher Scientific) (Ni et al., 2021). A reverse phase trap column together with a C18-reversed phase analytical column (Thermo Fisher Scientific) was used. The Q-Exactive mass spectrometer was operated in positive ion mode and MS data were dynamically acquired with top 10 data-dependent acquirements. The MASCOT engine (Matrix Science, London, United Kingdom; version 2.2) embedded into Proteome Discoverer 1.4 software was used for protein identification and quantification.

For the metabolomics analysis, metabolites were extracted using 400 μl of pre-cooled methanol/acetonitrile/water solution (2:2:1, v/v/v). After vortexing, ultrasonication, and incubation, the extracted mixture was centrifuged for 20 min at 14,000 × *g* and 4°C, and the supernatants were collected. The extracted metabolites were vacuum-dried, re-dissolved in 100 μl acetonitrile/water solution, and then transferred into sample vials for UHPLC-Q-TOF-MS analysis. Pooled quality control samples were simultaneously prepared for monitoring the stability and repeatability of the metabolomics platform. An Agilent 1290 Infinity LC system (Agilent Technologies, Santa Clara, CA, United States) equipped with an AB SCIEX Triple TOF 6600 mass spectrometer (AB SCIEX, Framingham, MA, United States) were used for global metabolite profiling. The injection volume of each sample was 2 μl, and the LC flow rate was set to 0.5 ml/min. The triple TOF 6600 was operated in both positive and negative ionization modes (Ni et al., 2021). The ion sources gas1 and gas2 were both 60 PSI, and the curtain gas was 30 PSI. The raw mass spectrometer files were converted to mzXML files using Proteo Wizard MS converter and processed using XCMS software for peak alignment and peak area extraction. Metabolite identification was achieved by mapping accurate mass and MS/MS spectra to the established reference library.

### Metabolomic analysis of fecal samples

The metabolomic analysis of fecal samples was performed as described for colon tissues.

### 16S rRNA amplicon sequencing analysis of fecal samples

Total bacterial DNA from fecal samples was extracted using the hexadecyl trimethyl ammonium bromide/SDS method, and the DNA concentration and purity were monitored on 1% agarose gels. 16S/18S rRNA genes were amplified by PCR in 15 μl of Phusion^®^High-Fidelity PCR Master Mix (New England Biolabs). PCR products were purified with AxyPrepDNA Gel Extraction Kit (AXYGEN). Sequencing libraries were generated with NEB Next^®^Ultra™DNA Library Prep Kit (NEB, United States) following the manufacturer’s instruction. The library was then sequenced on an Illumina NovaSeq 6000 platform, and fragments with 250 bp paired-end reads were generated (An et al., 2021). Fast length adjustment of short (FLASH) reads were applied to merge the paired-end reads from the original DNA fragments, which were assigned to each sample through the unique barcodes. Sequence analyses including operational taxonomic unit (OTU) cluster and species annotation were performed using the UPARSE software package (An et al., 2021).

### Cell experiments

HCT-116 cells (Manassas, VA) were cultured in Dulbecco’s modified Eagle’s medium supplemented with 10% fetal bovine serum. The cultured cells were treated with 200 μM peiminine solution or dimethyl sulfoxide as the control for 48 h. The treated cells were collected, stained with propidium iodide and annexin V, and analyzed using a BD Influx flow cytometer (BD Biosciences) to quantify apoptotic cells.

### Proteomics analysis of cell samples

A DIA-based proteomics approach was used to characterize the protein changes in peiminine-treated HCT-116 cells. Briefly, extracted proteins were quantified using a BCA protein assay kit (Bi Yuntian, Shanghai, China) and digested using the FASP method. The peptide concentrations were determined by measuring absorbance at 280 nm. The digested peptides were also analyzed in DDA mode as reported previously (Zheng et al., 2022). For DIA analysis, an Orbitrap Fusion Lumos mass spectrometer equipped with an EASY-nLC 1000 system (Thermo Fisher Scientific) were used. The full scan was performed at a resolution of 60,000 over an m/z range of 350–1500, and DIA scans were set at a resolution of 30,000. There were 45 variable DIA windows ranging from 350 to 1500 m/z. Spectronaut pulsar X 12.0 (Biognosys) software was used for protein identification and quantification.

### Data analysis

For proteomics data analysis, significantly changed proteins were identified by Student’s *t*-tests with a *p*-value ≤.05 (Ni et al., 2021). OmicsBean (http://www.omicsbean.com) was used for bioinformatics analysis including Gene Ontology (GO). For metabolomics analysis, the data were sum-normalized and then subjected to principal component analysis (PCA) and orthogonal partial least squares discrimination analysis (OPLS-DA) (Deng et al., 2020). Significantly altered metabolites were identified with variable importance by the projection (VIP) value > 1 and *p*-value ≤.05. Kyoto Encyclopedia of Genes and Genomes (KEGG) pathway enrichment analysis was performed using significantly changed proteins or metabolites. For the statistical analysis of 16S rRNA sequencing data, three metrics including chao1, Simpson, and Shannon were used to compute alpha diversity (An et al., 2021). To calculate beta diversity, clustering analysis and PCA were performed using the quantitative insights into microbial ecology (QIIME) software package. Principal coordinate analysis (PCoA) and the unweighted pair group method with arithmetic mean (UPGMA) clustering were also performed. Linear discriminant analysis (LDA) effect size (LEfSe) was used for the quantitative analysis of biomarkers within diversified groups.

## Results

### Proteomics study of colon tissues from YQSJ-treated mice

Pathological analyses were performed on colon tissues from the three groups of mice. The average number of colon tissue tumors per mouse in the CRC group was five, which was significantly higher than that in the YQSJ group (average two tumors per mouse) and control group (no tumors). The TMT-based proteomics approach was used to identify 7568 colon tissue proteins, among which 57 were significantly altered (*p* < .05) after YQSJ treatment, with fold changes ranging from .40 to 2.09 (Supplementary Table S1). There were 37 downregulated proteins and 20 upregulated proteins after YQSJ treatment. GO enrichment analysis of the 57 significantly changed proteins generated 227 significantly enriched terms (*p* < .05; Supplementary Table S2), of which 135 were related to biological processes, including cellular response to transforming growth factor beta stimulus, response to transforming growth factor beta, regulation of cytokine activity, and regulation of complement activation. There were 56 enriched molecular function terms, including phosphatidylinositol-4-phosphate binding, growth factor binding, sulfur compound binding, and transporter activity. Among 36 enriched terms in cellular component ontology, the top three terms were collagen type I trimer, collagen type III trimer, and cytoplasmic side of dendritic spine plasma membrane.

Pathway enrichment analysis of the significantly changed proteins identified 10 significantly enriched pathways that belonged to five different functional groups (Table 1). The Human Diseases group included three enriched pathways: amoebiasis, AGE-RAGE signaling in diabetic complications, and diabetic cardiomyopathy; the Organismal Systems group also had three pathways including protein digestion and absorption, relaxin signaling, and platelet activation; the Metabolism group included glycosaminoglycan biosynthesis-chondroitin sulfate/dermatan sulfate, and glycosaminoglycan biosynthesis-heparan sulfate/heparin; the Environmental Information Processing group included transforming growth factor (TGF)-beta signaling pathway related to signal transduction; and the Genetic Information Processing group was in enriched in the sulfur relay system pathway.

**TABLE 1:** KEGG pathway enrichment analysis of those 57 significantly changed protein.

Group	Map_Name	*p*-Value	Richfactor
Environmental Information Processing	TGF-beta signaling pathway	2.53E-02	0.06
Genetic Information Processing	Sulfur relay system	4.43E-02	0.17
Human Diseases	Amoebiasis	7.65E-03	0.06
	AGE-RAGE signaling pathway in diabetic complications	8.88E-03	0.05
	Diabetic cardiomyopathy	3.07E-02	0.03
Metabolism	Glycosaminoglycan biosynthesis-chondroitin sulfate/dermatan sulfate	4.43E-02	0.17
	Glycosaminoglycan biosynthesis-heparan sulfate/heparin	4.43E-02	0.17
Organismal Systems	Protein digestion and absorption	5.19E-03	0.06
	Relaxin signaling pathway	1.55E-02	0.04
	Platelet activation	2.21E-02	0.04

### Metabolomic changes of the colon tissues from YQSJ-treated mice

**FIGURE 1:**
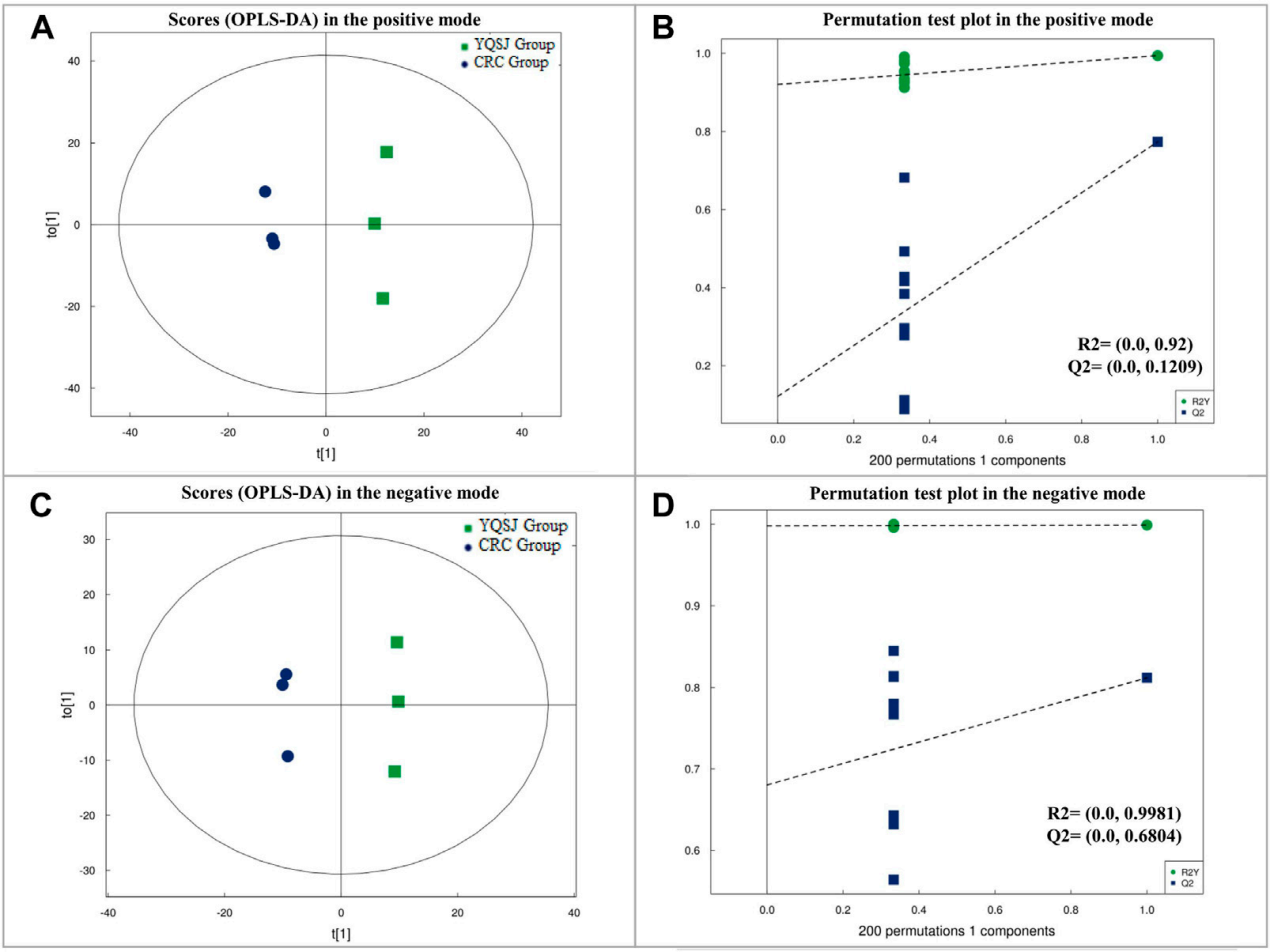
The results of OPLS-DA on metabolic data in the positive and negative modes, respectively. **(A)** Score plot in OPLS-DA model for colon tissue samples from CRC and YQSJ group in the positive mode. **(B)** Permutation test plot in OPLS-DA model for colon tissue samples from CRC and YQSJ group in the positive mode. **(C)** Score plot in OPLS-DA model for colon tissue samples from CRC and YQSJ group in the negative mode. **(D)** Permutation test plot in OPLS-DA model for colon tissue samples from CRC and YQSJ group in the negative mode.

**FIGURE 2:**
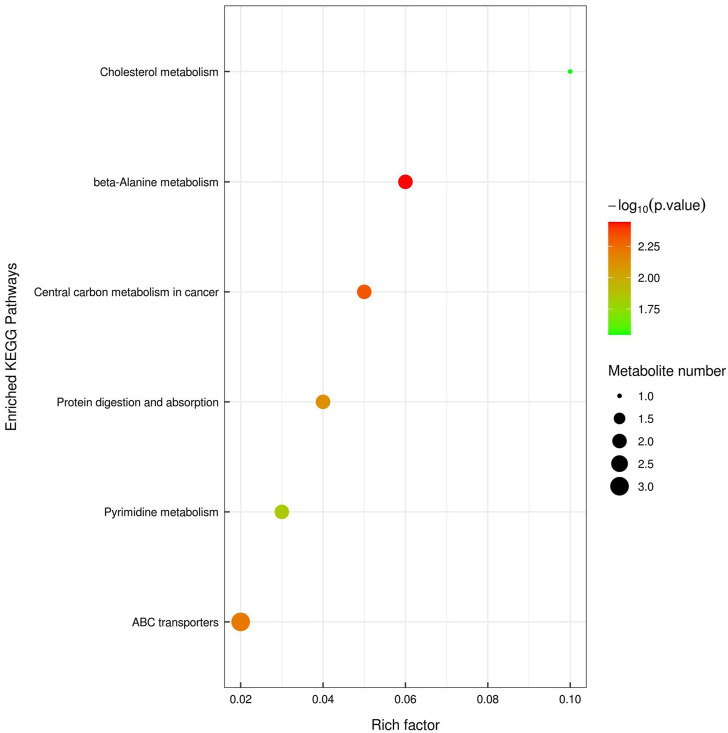
KEGG pathway enrichment analysis of those 37 significantly changed tissue metabolites. The color of the point represented *p*-value and its size indicated the number of different metabolites enriched. The value of rich factor represented the ratio between the number of different metabolites and the total metabolites in each pathway.

UPLC-TOF-MS/MS metabolomics profiling of the colon tissues used in the proteomics study detected 583 and 558 named metabolites in positive and negative modes, respectively. PCA analysis showed that the tissue samples from the CRC group were clearly separated from the samples from the YQSJ group under both ionization modes (Supplementary Figure S1). Supervised OPLS-DA was used to determine the metabolites responsible for the separation in the PCA (Figure 1). The results showed that 21 metabolites in positive ionization mode and 16 metabolites in negative ionization mode (VIP ≥1 and *p* ≤ .05) were significantly changed after YQSJ treatment. The ratios of the 37 metabolites between the CRC group and YQSJ group ranged from 0.51 to 9.61 (Supplementary Table S3). Most of the 37 metabolites (27 of 37) were decreased and belonged to lipids and lipid-like molecules. The metabolites increased by YQSJ treatment included pantothenate, cytosine, urea, and lignoceric acid. KEGG analysis of these 37 metabolites showed that they were enriched (*p* < .05) in six pathways including beta-Alanine metabolism, central carbon metabolism in cancer, ABC transporters, protein digestion and absorption, pyrimidine metabolism, and cholesterol metabolism (Figure 2).

### Metabolomics study of fecal samples from YQSJ-treated mice

**FIGURE 3:**
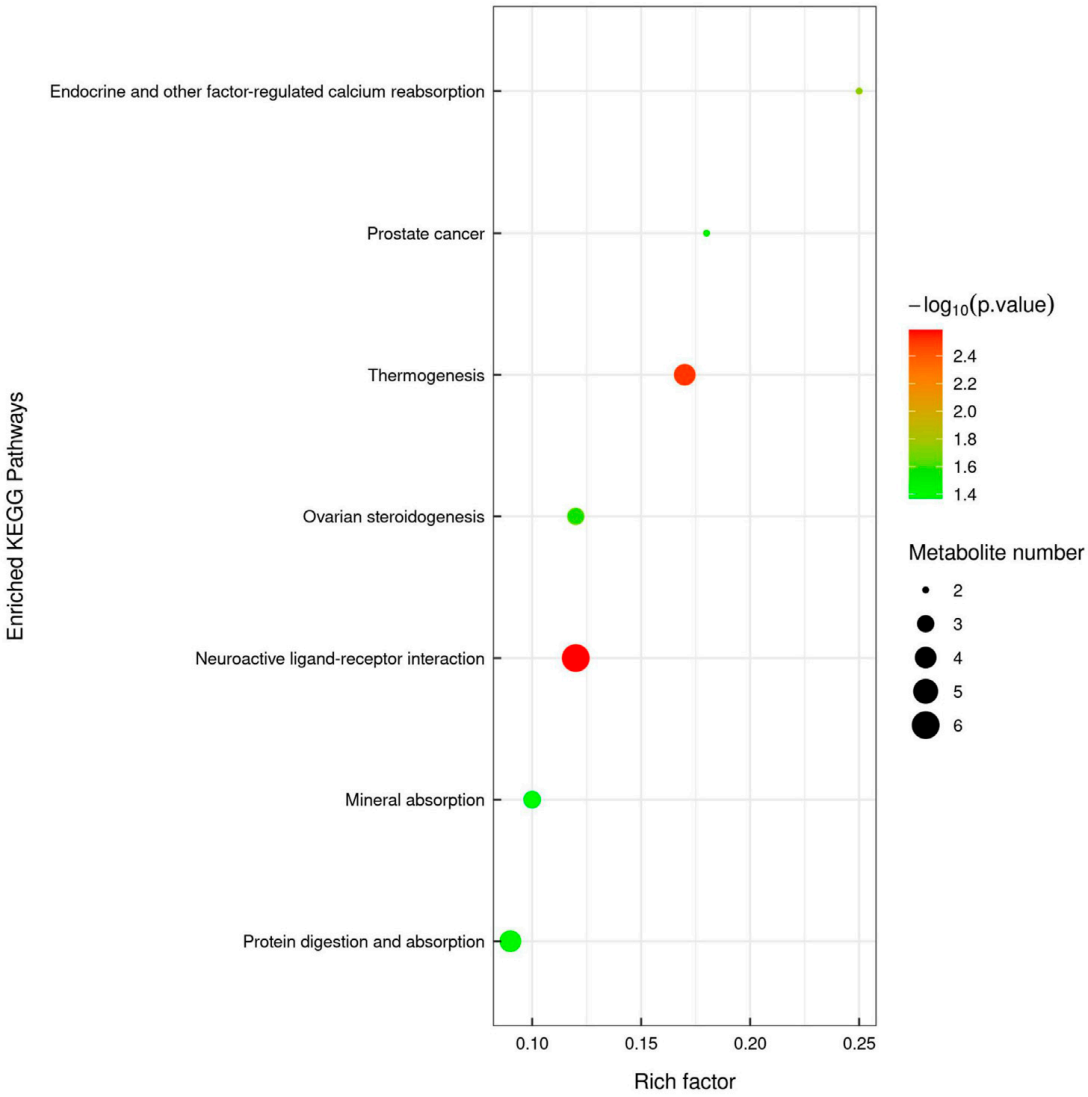
KEGG pathway enrichment analysis of those 121 significantly changed fecal metabolites. The color of the point represented *p*-value and its size indicated the number of different metabolites enriched. The value of rich factor represented the ratio between the number of different metabolites and the total metabolites in each pathway.

Non-targeted UPLC-TOF-MS/MS metabolomics analysis was performed to compare fecal samples from CRC and YQSJ mice. There were 1511 and 721 named metabolites determined in positive and negative mode, respectively. In addition, 227 metabolites were significantly changed (VIP ≥1 and *p* ≤ .05) at the endpoint in the CRC group (Supplementary Table S4). In the YQSJ group, 180 metabolites were significantly altered after YQSJ treatment (Supplementary Table S5). There were 59 common metabolites between the 227 and 180 changed metabolites in the two groups, and all except one (Pg 36:5) showed consistent changes. Hence, there were 121 significantly changed metabolites specific to YQSJ treatment (Supplementary Table S6), including 71 decreased and 50 increased metabolites with changes ranging from .05 to 325.14-fold. KEGG analysis returned seven significantly enriched (*p* < .05) pathways in the YQSJ group including neuroactive ligand-receptor interaction, thermogenesis, endocrine and other factor-regulated calcium reabsorption, protein digestion and absorption, and mineral absorption (Figure 3).

### 16S rRNA amplicon sequencing analysis of fecal samples from mice treated with YQSJ

**FIGURE 4:**
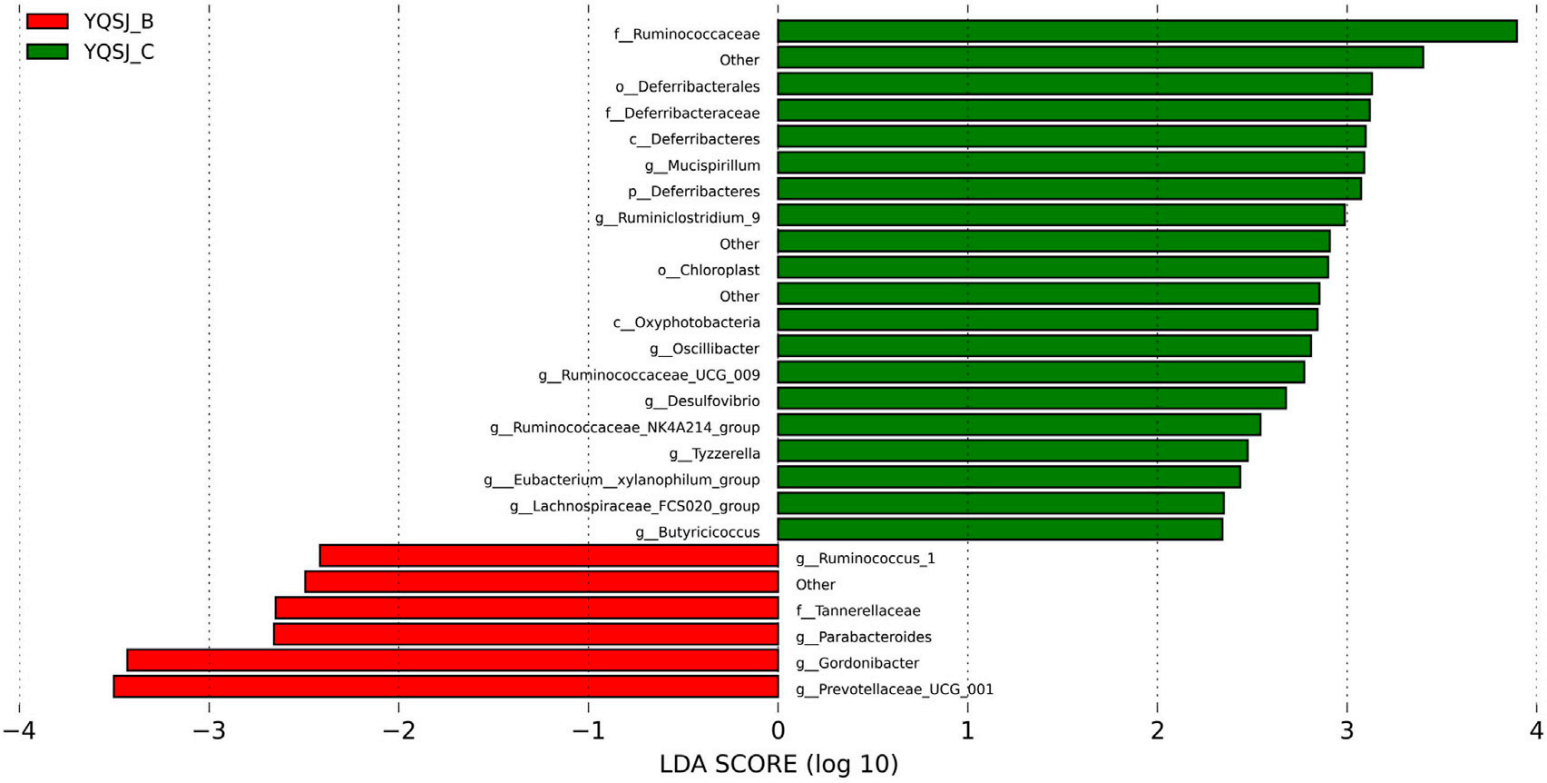
The LDA score in LEfSe analysis of bacterial community structure in YQSJ group. The distinct color represented the different stages in YQSJ group. The red color (YQSJ_B) indicated the mouse fecal samples were collected before treatment at day 27–28 in YQSJ group, while the green color (YQSJ_C) represented the mouse fecal samples were collected after treatment at day 66–67 (the end point) group. The “other” represented the flora that was not classified. The taxa were displayed (|LDA| > 2).

**FIGURE 5:**
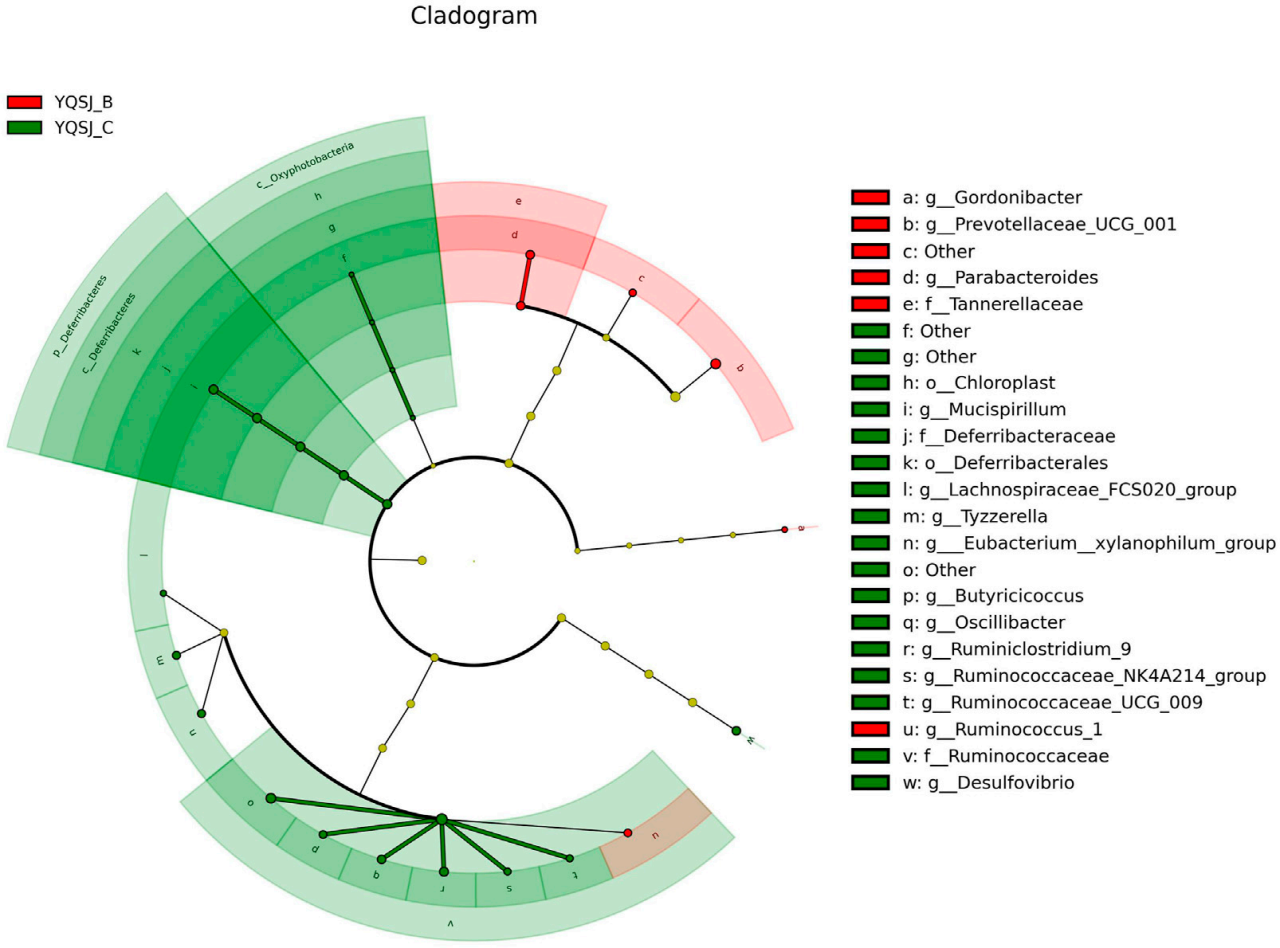
The cladogram in LEfSe analysis of bacterial community structure in YQSJ group. The branch chart displayed the differences in the fecal taxa. The red and green node represented the microbial group playing an important role in the red and green group, respectively. The red color (YQSJ_B) indicated that the mouse fecal samples were collected before treatment at day 27–28 in YQSJ group, while the green color (YQSJ_C) represented the mouse fecal samples were collected after treatment at day 66–67 (the end point) group. The “other” represented the flora that was not classified.

The top 11 most abundant fecal bacteria at the phylum level were Bacteroidetes, Firmicutes, Epsilonbacteraeaota, Tenericutes, Deferribacteres, Proteobacteria, Cyanobacteria, Patescibacteria, Actinobacteria, Verrucomicrobia, and Others (Supplementary Figure S2). Although Bacteroidetes and Firmicutes were dominant in both the CRC group and YQSJ group, Bacteroidetes were less abundant whereas Firmicutes were more abundant in YQSJ fecal samples than in the CRC group. At the genus level, *Lactobacillus* and Ruminococcaceae UCG-014 increased whereas Alloprevotella deceased after YQSJ treatment (Supplementary Figure S3). PCoA analysis showed significant changes in the gut microbiota structure following the development of CRC (Supplementary Figure S4). In the YQSJ group, the mouse gut microbiota was slightly changed after treatment, suggesting that YQSJ had a minimal impact on the general microbiota structure. LEfSe analysis identified the dominant bacteria in the CRC group (Model_B and Model_C) and the YQSJ group (YQSJ_B and YQSJ_C group) (Figures 4, 5; Supplementary Figures S5, S6). Parabacteroides, Tannerellaceae, Eubacterium__xylanophilum_group, Ruminococcus_1, and Prevotellaceae_UCG_001 were enriched in both CRC and YQSJ groups. Ruminococcus_1 and Prevotellaceae_UCG_001 exhibited opposite patterns of change, which may play important roles in tumor growth inhibition (Tang et al., 2020; Zhang et al., 2021). Prevotellaceae UCG-001, a beneficial bacterium, exerts anti-inflammatory effects and alleviates glucose and lipid metabolism disorders (Zhou et al., 2021). Ruminococcus are anti-inflammatory bacteria that produce the short chain fatty acid butyrate. The 16S rRNA sequencing results indicated that YQSJ ameliorates CRC development by regulating anti-inflammatory effects through the modulation of the intestinal microbial community.

### Proteomics study of peiminine-treated HCT-116 cells

**FIGURE 6:**
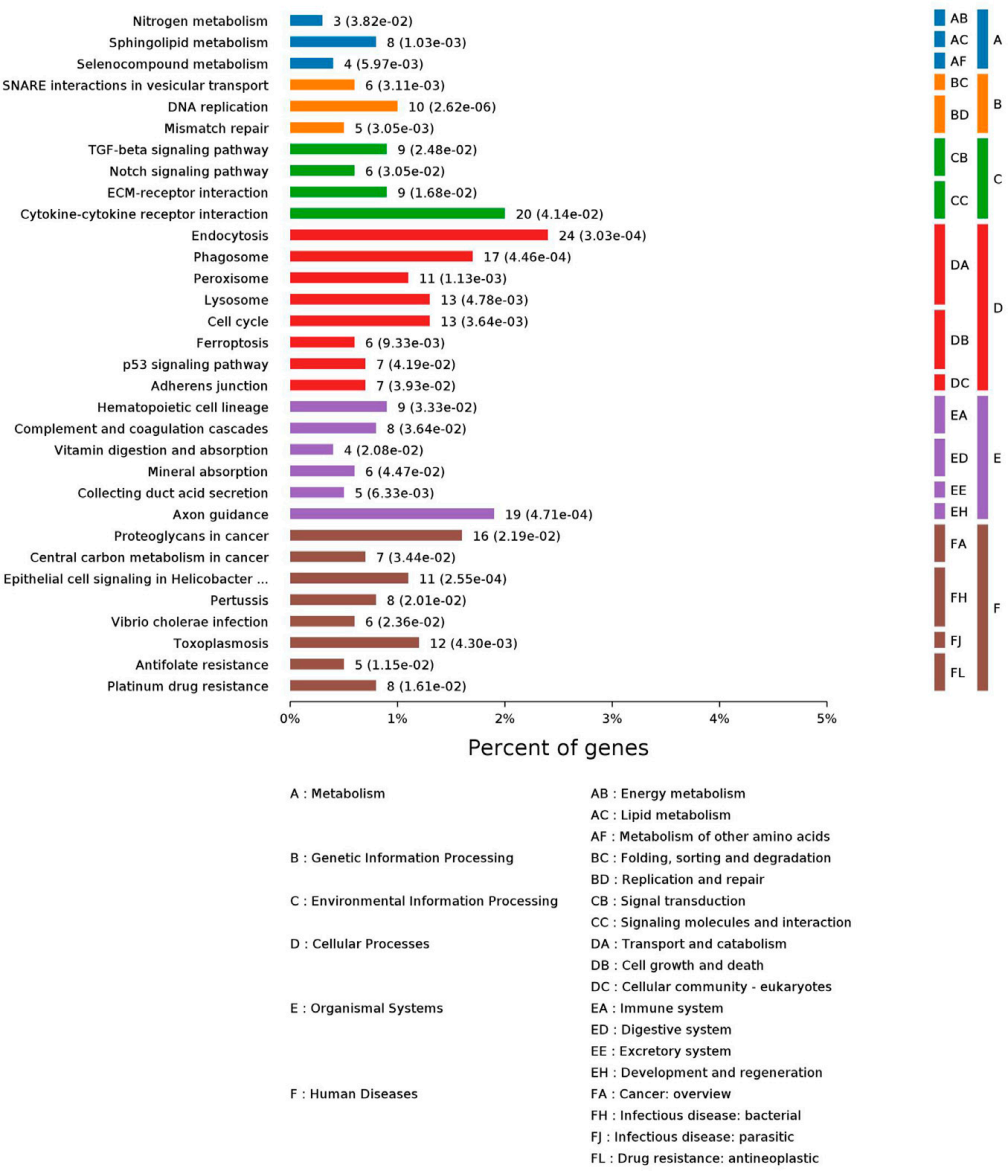
KEGG pathway enrichment analysis of those 1,073 significantly changed proteins. Each column represented one pathway, whose height indicated the number of different metabolites enriched.

Peiminine is the main and active component of the YQSJ formula. Consistent with previous data, peiminine treatment induced apoptosis and inhibited the growth of HCT-116 cells (Supplementary Figure S7). HCT-116 cells were treated with 200 μM of peiminine for 48 h and then collected for DIA-based proteomics study. Of 7152 proteins identified by the DIA proteomics method, 1073 were significantly altered (.02- to 359.44-fold) after peiminine treatment (Supplementary Table S7). There were 63 common proteins between the 1073 proteins and the significantly changed proteins in colon tissues after YQSJ treatment. Among the 63 common proteins, 32 including MAD2L1, MSH2, RFC4, and SQSTM1 showed the same pattern of change in HCT-116 cells and colon tissues: 13 downregulated and 19 upregulated proteins. GO enrichment analysis of these 1073 proteins generated 3281 enriched terms in the biological process category, 451 enriched terms in the cellular component category, and 178 enriched terms in the molecular function category (Supplementary Tables S8, S9, S10). The top ten enriched terms for each category are shown in Supplementary Figure S8, including cellular component organization or biogenesis, vesicle, extracellular exosome, and protein binding. KEGG pathway enrichment analysis of the 1073 significantly changed proteins identified 32 enriched pathways. The 32 pathways belonged to six different functional groups: Genetic information processing, human diseases, cellular processes, organismal systems, metabolism, and environmental information processing (Figure 6). The metabolism group included nitrogen metabolism, sphingolipid metabolism, and selenocompound metabolism. The genetic information processing group included DNA replication and mismatch repair pathways and others. The cellular processes group had eight significantly enriched pathways: endocytosis, phagosome, peroxisome, lysosome, cell cycle, ferroptosis, p53 signaling pathway, and adherens junction. The environmental information processing group consisted of ECM-receptor interaction, TGF-beta signaling pathway, Notch signaling pathway, and cytokine-cytokine receptor interaction. The organismal systems group had one pathway, complement and coagulation cascades.

## Discussion

TCM is effective in the treatment of CRC and can potentially delay cancer recurrence and suppress metastasis (Ge et al., 2022). We previously showed that YQSJ formula in combination with mFOLFOX6 postoperative adjuvant chemotherapy improves the survival rate of advanced CRC patients and decreases the incidence of side effects (Zhang et al., 2015; Zheng et al., 2022). The main active component of the YQSJ formula, peiminine, increases autophagic flux in HCT-116 cells *via* the AMPK pathway by SQSTM1. TCM has multi-component and multi-target characteristics. Hence, we used multi-omics technologies including proteomics, metabolomics, and 16S rRNA amplicon sequencing to comprehensively characterize the antitumor mechanism of the YQSJ formula and peiminine in the current study.

Proteomics analysis of peiminine-treated HCT-116 cells indicated that peiminine induced ferroptosis. The ferroptosis process has recently attracted substantial attention in cancer studies because ferroptotic damage favors tumor growth by triggering inflammation-related immunosuppression in the tumor microenvironment (Chen et al., 2021). Ferroptosis affects the response to chemotherapy and immunotherapy. For example, metallothionein-1G promotes sorafenib resistance by inhibiting ferroptosis (Sun et al., 2016). We identified six proteins involved in ferroptosis that were significantly changed in response to peiminine treatment: NCOA4, FTH1, SLC7A11, SLC39A14, STEAP3, and TFRC. NCOA4, a key molecule for promoting ferroptosis, was upregulated by more than 26-fold after peiminine treatment in HCT-116 cells (Mou et al., 2021). In a previous report, we showed that several changed metabolites including glutamine, cysteine, and six peptide compounds were related to ferroptosis (Zheng et al., 2017). Taken together, these results suggest that the induction of ferroptosis by peiminine plays a key role in reversing chemoresistance in advanced CRC patients. Similar to other natural products, including artesunate and icariin, peiminine may exert its therapeutic effect against CRC by promoting DNA damage repair (Li et al., 2008; Li et al., 2019). In HCT-116 cells, peiminine affected certain cellular processes and genetic information processes such as cell cycle, DNA replication, and mismatch repair. Peiminine significantly downregulated the expression of MSH2 and RFC4, which are involved in these processes and are associated with various cancers including CRC (Fishel et al., 1993; Xiang et al., 2014).

The anticancer effects of peiminine may also be related to its anti-inflammatory activity. Peiminine induces apoptosis in HepG2 cells to evade local inflammatory reactions and tissue damage (Chao et al., 2019). Peiminine downregulates the expression of inflammatory cytokines in cancer cells, decreasing the levels of COX-2, TNF-α, IL-1β, as well as suppressing NF-κB signaling (Lim et al., 2018). In both proteomics studies of HCT-116 cells and colon tissue, the TGF-β signaling pathway was significantly enriched after peiminine or YQSJ formula treatment. TGF-β acts as a tumor promoter, and TGF-β signaling has been widely targeted in cancer therapy (Larson et al., 2020). Peiminine also regulated two inflammation-related pathways: cytokine-cytokine receptor interaction and complement and coagulation cascades. Cytokine-cytokine interactions play an important role in modulating inflammatory and immunological responses in various diseases including cancer (Saadi et al., 2010). Complement and coagulation cascades play a crucial role in regulating thrombo-inflammatory processes in cancer development (Bauer et al., 2022). Several other important inflammation-related pathways including sphingolipid metabolism and cholesterol metabolism were also significantly changed after peiminine or YQSJ formula treatment. Dysregulation of sphingolipid metabolism and cholesterol metabolism is associated with CRC progression (Ryland et al., 2011; Huang et al., 2020).

The human gut microbiota is emerging as a critical player in human health; it can affect the host immune system and plays important roles in CRC development (Pothuraju et al., 2021). Similar to dietary interventions, TCM can modulate the gut microbiome and improve inflammatory/metabolic phenotypes, exerting protective effects against CRC (Zhang et al., 2020). Lv et al. (2019) showed that Gegen Qinlian decoction improves the effect of PD-1 blockade in CRC by remodeling the gut microbiota and the corresponding tumor microenvironment. In this study, we found that the YQSJ formula regulated the composition of the gut microbiota in AOM/DSS-induced CRC mice and delayed the development of CRC. YQSJ increases the levels of beneficial bacteria such as Ruminococcus_1 and Prevotellaceae_UCG_001 (Wang et al., 2021). Firmicutes, which play important roles in maintaining gut homeostasis and thus positively affect human health, were significantly increased after YQSJ treatment (Sun et al., 2022). The protein digestion and absorption pathway was significantly enriched by YQSJ treatment, as indicated by proteomics, metabolomics, and 16S rRNA amplicon sequencing results. These findings suggest that the protein digestion and absorption pathway is a target of YQSJ formula and could be a promising target for the treatment of CRC, as indicated by its involvement in various types of cancer including papillary thyroid cancer, gastric cancer, and breast cancer.

## Conclusion

In this multi-omics study, we identified molecular and microbiota changes induced by YQSJ or its main active component peiminine. Key regulatory elements or pathways involved in the therapeutic effect of YQSJ on CRC included DNA damage repair, ferroptosis, and several inflammation-related pathways. The beneficial effects of YQSJ formula on CRC were possibly mediated by the modulation of the gut microbiome to increase the relative contents of beneficial bacteria. The present findings provide new insight into the antitumor mechanism of YQSJ/peiminine and expand our understanding of the therapeutic application of YQSJ in CRC. The present integrative proteomics and metabolomics analyses identified proteins and metabolites that were significantly altered by YQSJ/peiminine treatment in CRC; however, additional studies are needed to explore the precise mechanisms underlying these changes by manipulating specific protein or metabolite targets in animal models or cell experiments.

## Data Availability

The datasets presented in this study can be found in online repositories. The names of the repository/repositories and accession number(s) can be found below: ProteomeXchange (http://proteomecentral.proteomexchange.org/cgi/GetDataset), PXD037110; Genome Sequence Archive in National Genomics Data Center, China National Center for Bioinformation/Beijing Institute of Genomics, Chinese Academy of Sciences (https://ngdc.cncb.ac.cn/gsa/), CRA008830.

## Supplementary material

SUPPLEMENTARY FIGURE S1 PCA scores plot generated from the detected features across all colon tissues samples in the positive (**A**) and negative (**B**) modes, respectively.

SUPPLEMENTARY FIGURE S2 The top 11 species in abundance at the phylum level for the tested fecal samples in CRC group and YQSJ group.

SUPPLEMENTARY FIGURE S3 The top 11 species in abundance at the genus level for the tested fecal samples in CRC group and YQSJ group.

SUPPLEMENTARY FIGURE S4 PCoA analysis of bacterial community structure in CRC group and YQSJ group.

SUPPLEMENTARY FIGURE S5 The LDA score in LEfSe analysis of bacterial community structure in CRC group.

SUPPLEMENTARY FIGURE S6 The cladogram in LEfSe analysis of bacterial community structure in CRC group.

SUPPLEMENTARY FIGURE S7 Peiminine induced apoptosis in HCT-116 cells by using flow cytometry.

SUPPLEMENTARY FIGURE S8 The top ten enriched terms for each category in GO enrichment analysis based on 1073 significantly changed proteins.

## Abbreviations

UPLC-TOF-MS/MS, Ultra high performance liquid chromatography coupled with quadrupole-time-of-flight mass spectrometry; UHPLC-Q-Exactive Orbitrap MS, Ultra high performance liquid chromatography equipped with quadrupole-exactive orbitrap mass spectrometry; TMT, Tandem mass tag; DIA, Data independent acquisition; PCA, Principal component analysis; OPLS-DA, Orthogonal partial least squares discrimination analysis; QC, Quality control; YQSJ, Yiqi Sanjie, CRC, Colorectal cancer; TCMs, Traditional Chinese Medicines

## References

[B1] An G. Zhang Y. Fan L. Chen J. Wei M. Li C. (2021). Integrative analysis of vaginal microorganisms and serum metabolomics in rats with estrous cycle disorder induced by long-term heat exposure based on 16S rDNA gene sequencing and LC/MS-based metabolomics. Front. Cell. Infect. Microbiol. 11, 595716. 10.3389/fcimb.2021.595716 33738264 PMC7962411

[B2] Bauer A. T. Gorzelanny C. Gebhardt C. Pantel K. Schneider S. W. (2022). Interplay between coagulation and inflammation in cancer: Limitations and therapeutic opportunities. Cancer Treat. Rev. 102, 102322. 10.1016/j.ctrv.2021.102322 34922151

[B3] Catalano F. Borea R. Puglisi S. Boutros A. Gandini A. Cremante M. (2022). Targeting the DNA damage response pathway as a novel therapeutic strategy in colorectal cancer. Cancers 14, 1388. 10.3390/cancers14061388 35326540 PMC8946235

[B4] Chao X. Wang G. Tang Y. Dong C. Li H. Wang B. (2019). The effects and mechanism of peiminine-induced apoptosis in human hepatocellular carcinoma HepG2 cells. PLoS One 14, e0201864. 10.1371/journal.pone.0201864 30615617 PMC6322737

[B5] Chen T. Chen X. Zhang S. Zhu J. Tang B. Wang A. (2021). The Genome sequence archive family: Toward explosive data growth and diverse data types. Genomics Proteomics Bioinforma. 19, 578–583. 10.1016/j.gpb.2021.08.001 PMC9039563 34400360

[B6] Chen X. Kang R. Kroemer G. Tang D. (2021). Broadening horizons: The role of ferroptosis in cancer. Nat. Rev. Clin. Oncol. 18, 280–296. 10.1038/s41571-020-00462-0 33514910

[B7] Ciardiello F. Ciardiello D. Martini G. Napolitano S. Tabernero J. Cervantes A. (2022). Clinical management of metastatic colorectal cancer in the era of precision medicine. Ca. Cancer J. Clin. 72, 372–401. 10.3322/caac.21728 35472088

[B8] CNCB-NGDC Members and Partners (2022). Database resources of the national Genomics data center, China national center for bioinformation in 2022. Nucleic Acids Res. 50, 27–38. 10.1093/nar/gkab951 PMC8728233 34718731

[B9] Deng W. Rao J. Chen X. Li D. Zhang Z. Liu D. (2020). Metabolomics study of serum and urine samples reveals metabolic pathways and biomarkers associated with pelvic organ prolapse. J. Chromatogr. B Anal. Technol. Biomed. Life Sci. 1136, 121882. 10.1016/j.jchromb.2019.121882 31809960

[B10] El Bali M. Bakkach J. Bennani Mechita M. (2021). Colorectal cancer: From genetic landscape to targeted therapy. J. Oncol. 2021, 9918116. 10.1155/2021/9918116 34326875 PMC8277501

[B11] Feng W. Liu J. Huang L. Tan Y. Peng C. (2021). Gut microbiota as a target to limit toxic effects of traditional Chinese medicine: Implications for therapy. Biomed. Pharmacother. 133, 111047. 10.1016/j.biopha.2020.111047 33378954

[B12] Fishel R. Lescoe M. K. Rao M. R. Copeland N. G. Jenkins N. A. Garber J. (1993). The human mutator gene homolog MSH2 and its association with hereditary nonpolyposis colon cancer. Cell 75, 1027–1038. 10.1016/0092-8674(93)90546-3 8252616

[B13] Gao Y. Chen S. Sun J. Su S. Yang D. Xiang L. (2021). Traditional Chinese medicine may be further explored as candidate drugs for pancreatic cancer: A review. Phytother. Res. 35, 603–628. 10.1002/ptr.6847 32965773

[B14] Ge H. Xu C. Chen H. Liu L. Zhang L. Wu C. (2022). Traditional Chinese medicines as effective reversals of epithelial-mesenchymal transition induced-metastasis of colorectal cancer: Molecular targets and mechanisms. Front. Pharmacol. 13, 842295. 10.3389/fphar.2022.842295 35308223 PMC8931761

[B15] Heinrich M. Jalil B. Abdel-Tawab M. Echeverria J. Kulic Z. Mcgaw L. J. (2022). Best Practice in the chemical characterisation of extracts used in pharmacological and toxicological research-The ConPhyMP-Guidelines. Front. Pharmacol. 13, 953205. 10.3389/fphar.2022.953205 36176427 PMC9514875

[B16] Huang B. Song B. Xu C. (2020). Cholesterol metabolism in cancer: Mechanisms and therapeutic opportunities. Nat. Metab. 2, 132–141. 10.1038/s42255-020-0174-0 32694690

[B17] Larson C. Oronsky B. Carter C. A. Oronsky A. Knox S. J. Sher D. (2020). TGF-Beta: A master immune regulator. Expert Opin. Ther. Targets 24, 427–438. 10.1080/14728222.2020.1744568 32228232

[B18] Li J. X. Li R. Z. Sun A. Zhou H. Neher E. Yang J. S. (2021). Metabolomics and integrated network pharmacology analysis reveal Tricin as the active anti-cancer component of Weijing decoction by suppression of PRKCA and sphingolipid signaling. Pharmacol. Res. 171, 105574. 10.1016/j.phrs.2021.105574 34419228

[B19] Li N. Wang J. Wang X. Sun J. Li Z. (2019). Icariin exerts a protective effect against d-galactose induced premature ovarian failure via promoting DNA damage repair. Biomed. Pharmacother. 118, 109218. 10.1016/j.biopha.2019.109218 31330441

[B20] Li P. C. Lam E. Roos W. P. Zdzienicka M. Z. Kaina B. Efferth T. (2008). Artesunate derived from traditional Chinese medicine induces DNA damage and repair. Cancer Res. 68, 4347–4351. 10.1158/0008-5472.CAN-07-2970 18519695

[B21] Li Z. Fei Z. Gao L. (2021a). Traditional Chinese medicine and lung cancer-From theory to practice. Biomed. Pharmacother. 137, 111381. 10.1016/j.biopha.2021.111381 33601147

[B22] Li Z. Yin D. F. Wang W. Zhang X. W. Zhou L. J. Yang J. (2021b). Efficacy of Yiqi Jianpi anti-cancer prescription combined with chemotherapy in patients with colorectal cancer after operation. World J. Clin. Cases 9, 9869–9877. 10.12998/wjcc.v9.i32.9869 34877325 PMC8610922

[B23] Li-Weber M. (2013). Targeting apoptosis pathways in cancer by Chinese medicine. Cancer Lett. 332, 304–312. 10.1016/j.canlet.2010.07.015 20685036

[B24] Lim J. M. Lee B. Min J. H. Kim E. Y. Kim J. H. Hong S. (2018). Effect of peiminine on DNCB-induced atopic dermatitis by inhibiting inflammatory cytokine expression *in vivo* and *in vitro* . Int. Immunopharmacol. 56, 135–142. 10.1016/j.intimp.2018.01.025 29414643

[B25] Luo H. Sun S. J. Wang Y. Wang Y. L. (2020). Revealing the sedative-hypnotic effect of the extracts of herb pair Semen Ziziphi spinosae and Radix Polygalae and related mechanisms through experiments and metabolomics approach. BMC Complement. Med. Ther. 20, 206. 10.1186/s12906-020-03000-8 32615973 PMC7330955

[B26] Lv J. Jia Y. Li J. Kuai W. Li Y. Guo F. (2019). Gegen Qinlian decoction enhances the effect of PD-1 blockade in colorectal cancer with microsatellite stability by remodelling the gut microbiota and the tumour microenvironment. Cell Death Dis. 10, 415. 10.1038/s41419-019-1638-6 31138779 PMC6538740

[B27] Ma J. Deng Y. Zhang M. Yu J. (2022). The role of multi-omics in the diagnosis of COVID-19 and the prediction of new therapeutic targets. Virulence 13, 1101–1110. 10.1080/21505594.2022.2092941 35801633 PMC9272836

[B28] Mou Y. Wu J. Zhang Y. Abdihamid O. Duan C. Li B. (2021). Low expression of ferritinophagy-related NCOA4 gene in relation to unfavorable outcome and defective immune cells infiltration in clear cell renal carcinoma. BMC Cancer 21, 18. 10.1186/s12885-020-07726-z 33402128 PMC7786469

[B29] Ni T. Xu S. Wei Y. Li T. Jin G. Deng W. W. (2021). Understanding the promotion of withering treatment on quality of postharvest tea leaves using UHPLC-orbitrap-MS metabolomics integrated with TMT-Based proteomics. LWT 147, 111614. 10.1016/j.lwt.2021.111614

[B30] Pothuraju R. Chaudhary S. Rachagani S. Kaur S. Roy H. K. Bouvet M. (2021). Mucins, gut microbiota, and postbiotics role in colorectal cancer. Gut Microbes 13, 1974795. 10.1080/19490976.2021.1974795 34586012 PMC8489937

[B31] Ryland L. K. Fox T. E. Liu X. Loughran T. P. Kester M. (2011). Dysregulation of sphingolipid metabolism in cancer. Cancer Biol. Ther. 11, 138–149. 10.4161/cbt.11.2.14624 21209555

[B32] Saadi A. Shannon N. B. Lao-Sirieix P. O'Donovan M. Walker E. Clemons N. J. (2010). Stromal genes discriminate preinvasive from invasive disease, predict outcome, and highlight inflammatory pathways in digestive cancers. Proc. Natl. Acad. Sci. U. S. A. 107, 2177–2182. 10.1073/pnas.0909797107 20080664 PMC2836667

[B33] Sun X. Niu X. Chen R. He W. Chen D. Kang R. (2016). Metallothionein-1G facilitates sorafenib resistance through inhibition of ferroptosis. Hepatology 64, 488–500. 10.1002/hep.28574 27015352 PMC4956496

[B34] Sun Y. Zhang S. Nie Q. He H. Tan H. Geng F. (2022). Gut firmicutes: Relationship with dietary fiber and role in host homeostasis. Crit. Rev. Food Sci. Nutr. 12, 1–16. 10.1080/10408398.2022.2098249 35822206

[B35] Tang R. Z. Li Z. Z. Hu D. Kanwal F. Yuan C. B. Mustaqeem M. (2021). Sanjie yiliu formula inhibits colorectal cancer growth by suppression of proliferation and induction of apoptosis. ACS Omega 6, 7761–7770. 10.1021/acsomega.0c05565 33778287 PMC7992181

[B36] Tang S. Xin Y. Ma Y. Xu X. Zhao S. Cao J. (2020). Screening of microbes associated with swine growth and fat deposition traits across the intestinal tract. Front. Microbiol. 11, 586776. 10.3389/fmicb.2020.586776 33178171 PMC7596661

[B37] Visal T. H. den Hollander P. Cristofanilli M. Mani S. A. (2022). Circulating tumour cells in the-omics era: How far are we from achieving the ‘singularity. Br. J. Cancer 127, 173–184. 10.1038/s41416-022-01768-9 35273384 PMC9296521

[B38] Wang Y. Zhang X. Li J. Zhang Y. Guo Y. Chang Q. (2021). Sini decoction ameliorates colorectal cancer and modulates the composition of gut microbiota in mice. Front. Pharmacol. 12, 609992. 10.3389/fphar.2021.609992 33776762 PMC7991589

[B39] Xiang J. Fang L. Luo Y. Yang Z. Liao Y. Cui J. (2014). Levels of human replication factor C4, a clamp loader, correlate with tumor progression and predict the prognosis for colorectal cancer. J. Transl. Med. 12, 320. 10.1186/s12967-014-0320-0 25407051 PMC4256821

[B40] Xu W. Li B. Xu M. Yang T. Hao X. (2022). Traditional Chinese medicine for precancerous lesions of gastric cancer: A review. Biomed. Pharmacother. 146, 112542. 10.1016/j.biopha.2021.112542 34929576

[B41] Xu X. Zhang J. Zhang Z. Wang M. Liu Y. Li X. (2020). Systems pharmacology in combination with proteomics reveals underlying mechanisms of Xihuang pill against triple-negative breast cancer. Bioengineered 11, 1170–1188. 10.1080/21655979.2020.1834726 33092442 PMC8291799

[B42] Yang Z. Zhang Q. Yu L. Zhu J. Cao Y. Gao X. (2021). The signaling pathways and targets of traditional Chinese medicine and natural medicine in triple-negative breast cancer. J. Ethnopharmacol. 264, 113249. 10.1016/j.jep.2020.113249 32810619

[B43] Zhang A. H. Zhou X. H. Zhao H. W. Zou S. Y. Ma C. W. Liu Q. (2017). Metabolomics and proteomics technologies to explore the herbal preparation affecting metabolic disorders using high resolution mass spectrometry. Mol. Biosyst. 13, 320–329. 10.1039/c6mb00677a 28045158

[B44] Zhang R. Gao X. Bai H. Ning K. (2020). Traditional Chinese medicine and gut microbiome: Their respective and concert effects on healthcare. Front. Pharmacol. 11, 538. 10.3389/fphar.2020.00538 32390855 PMC7188910

[B45] Zhang Z. Bing W. U. Liu Z. Y. Si F. Zheng Z. (2015). Clinical study on the combination treatment of qi supplied and stagnation resolved therapy with Mfolfox6 postoperative adjuvant chemotherapy for the stage Ⅲ colon cancer. World J. Integr. Traditional West. Med. 10, 995–998.

[B46] Zhang Z. M. Yang L. Wan Y. Liu C. Jiang S. Shang E. X. (2021). Integrated gut microbiota and fecal metabolomics reveal the renoprotective effect of Rehmanniae Radix Preparata and Corni Fructus on adenine-induced CKD rats. J. Chromatogr. B Anal. Technol. Biomed. Life Sci. 1174, 122728. 10.1016/j.jchromb.2021.122728 33975272

[B47] Zheng Z. Wei Q. Wan X. Zhong X. Liu L. Zeng J. (2022). Correlation analysis between trace elements and colorectal cancer metabolism by integrated serum Proteome and metabolome. Front. Immunol. 13, 921317. 10.3389/fimmu.2022.921317 35720415 PMC9201339

[B48] Zheng Z. Xu L. Zhang S. Li W. Tou F. He Q. (2017). Peiminine inhibits colorectal cancer cell proliferation by inducing apoptosis and autophagy and modulating key metabolic pathways. Oncotarget 8, 47619–47631. 10.18632/oncotarget.17411 28496003 PMC5564592

[B49] Zhou X. Zhang B. Zhao X. Lin Y. Wang J. Wang X. (2021). Chlorogenic acid supplementation ameliorates hyperuricemia, relieves renal inflammation, and modulates intestinal homeostasis. Food Funct. 12, 5637–5649. 10.1039/d0fo03199b 34018499

