# Supplementary material for: Integrative analysis and identification of key elements and pathways regulated by Traditional Chinese Medicine (Yiqi Sanjie formula) in colorectal cancer
Source: Front Pharmacol. 2022 Dec 13;13:1090599. doi: 10.3389/fphar.2022.1090599 (PMC9792787; doi:10.3389/fphar.2022.1090599)
Supplement: Supplementary file 1 [file Presentation1.PPTX]

## Slide 1
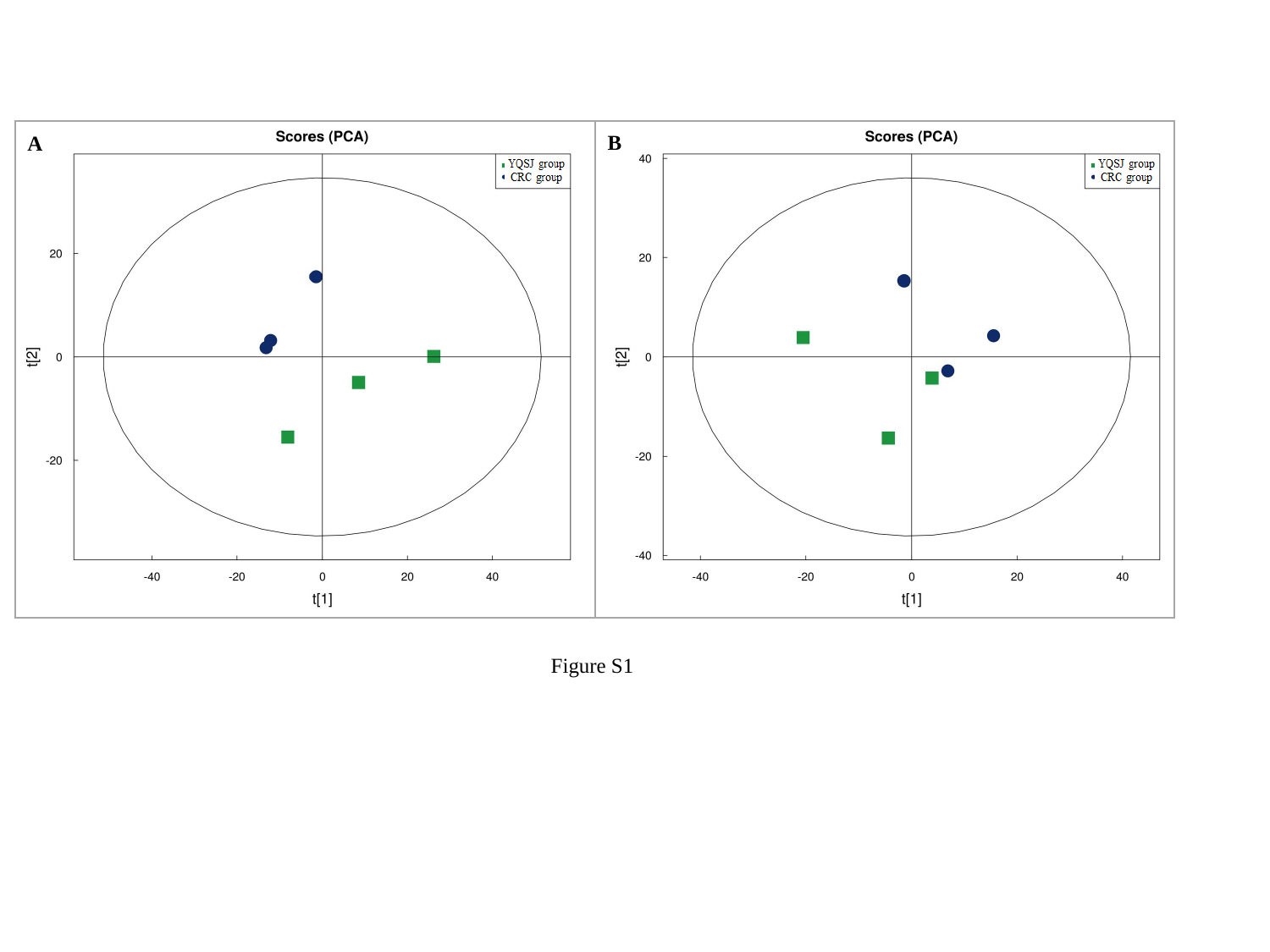

B
A
Figure S1

## Slide 2
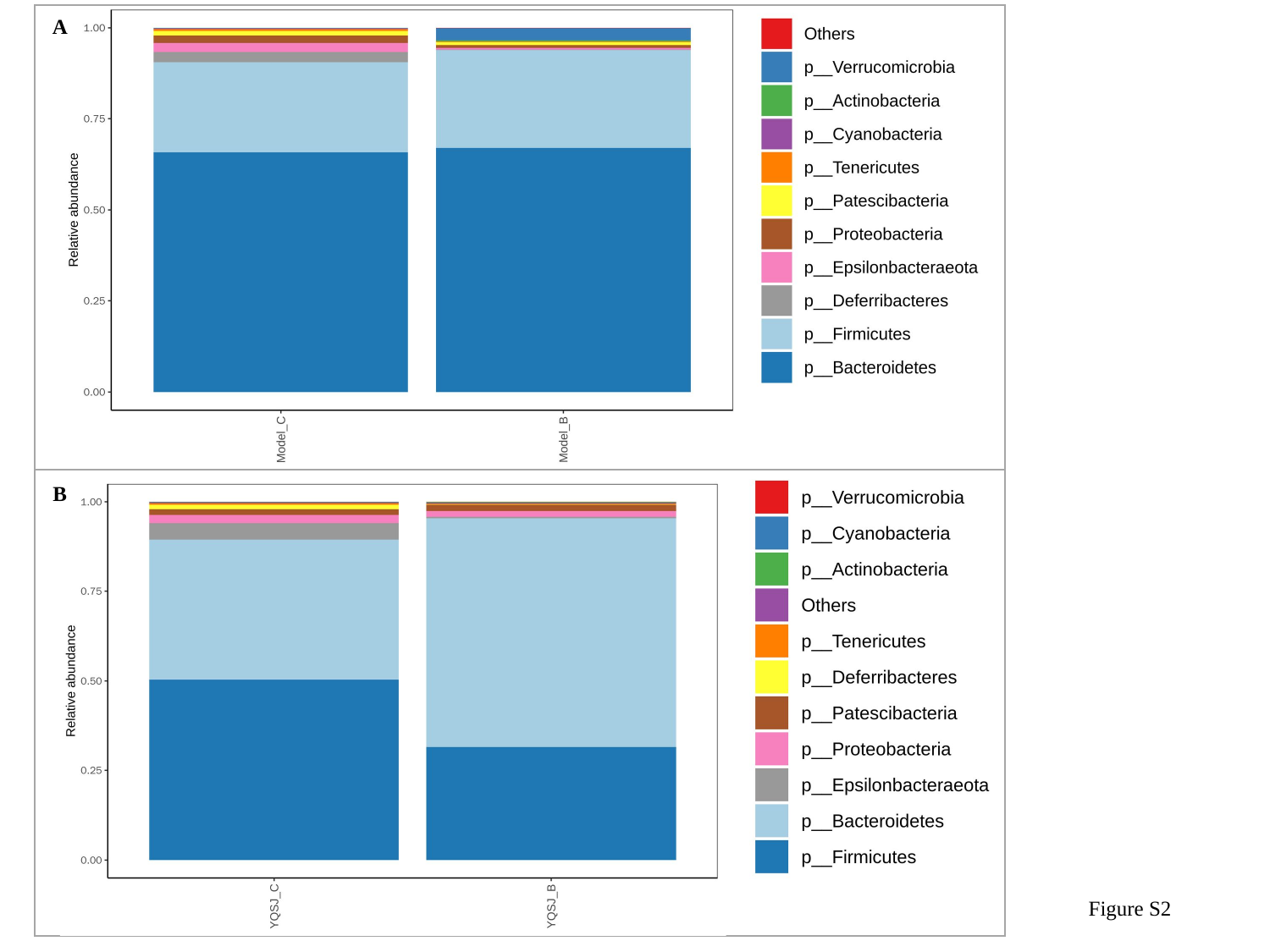

A
B
Figure S2

## Slide 3
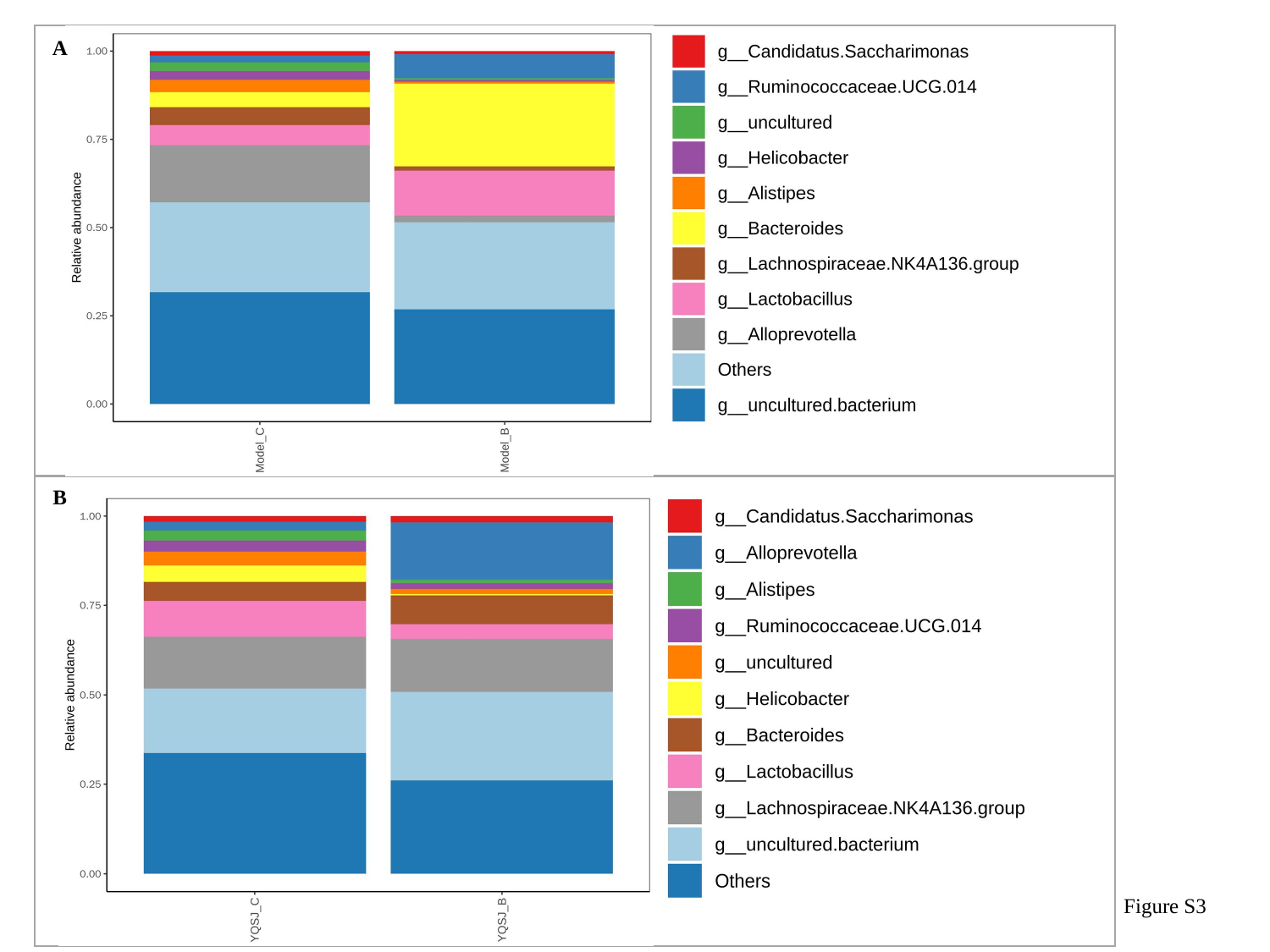

A
B
Figure S3

## Slide 4
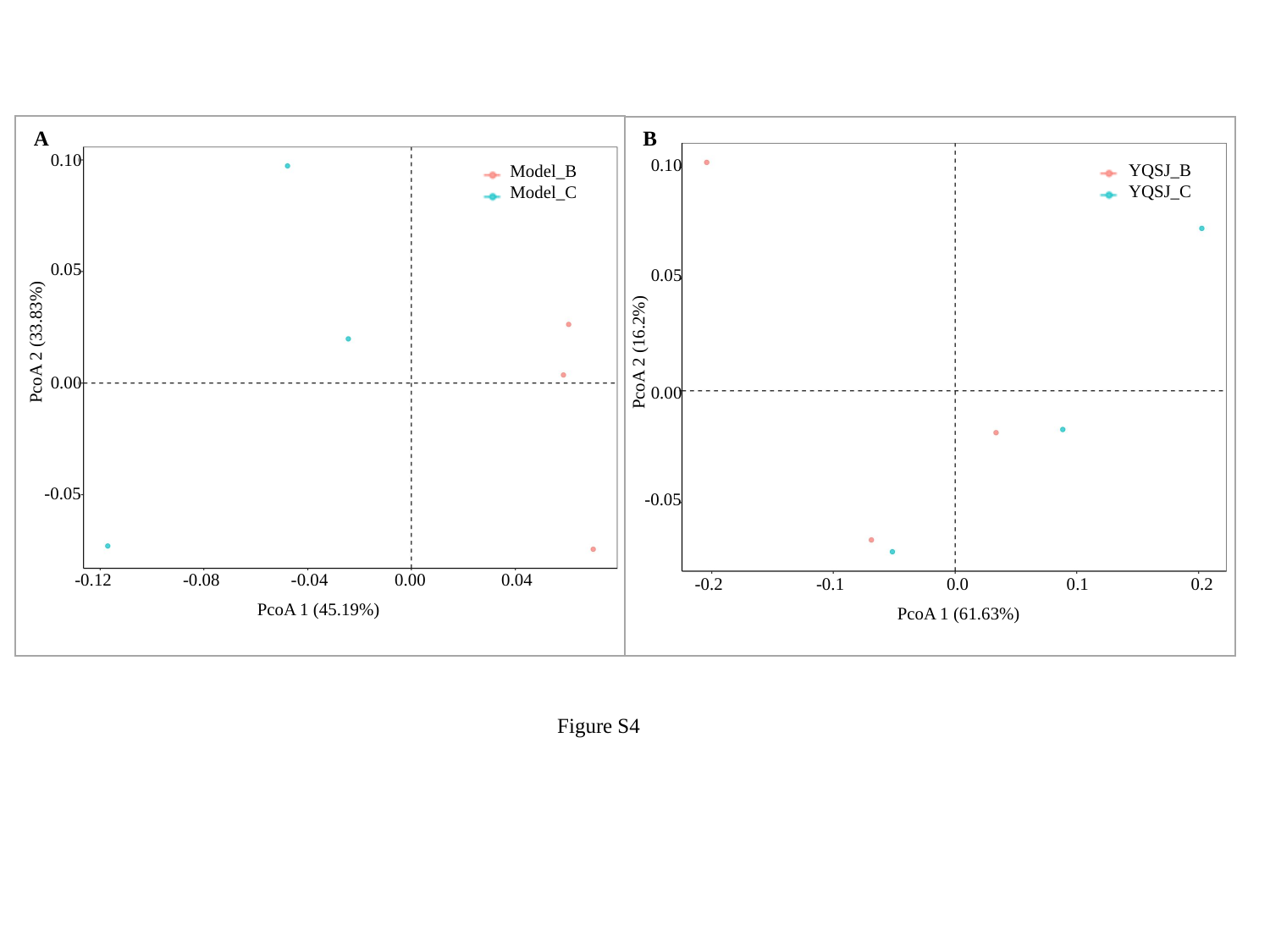

A
B
0.10
0.05
0.00
-0.05
0.10
0.05
PcoA 2 (16.2%)
0.00
-0.05
YQSJ_B
YQSJ_C
Model_B
Model_C
PcoA 2 (33.83%)
-0.12 -0.08 -0.04 0.00 0.04
-0.2 -0.1 0.0 0.1 0.2
PcoA 1 (45.19%)
PcoA 1 (61.63%)
Figure S4

## Slide 5
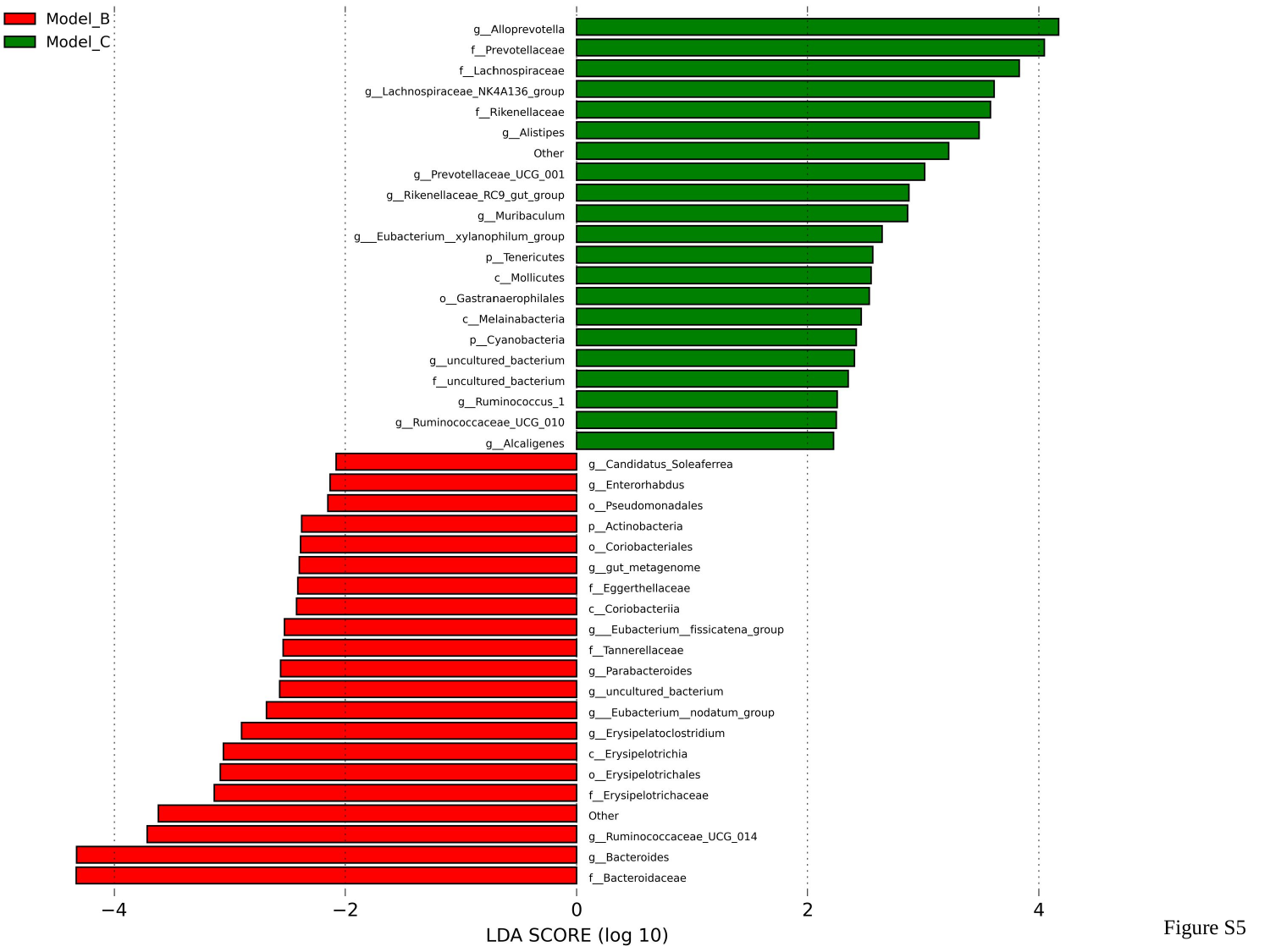

Figure S5

## Slide 6
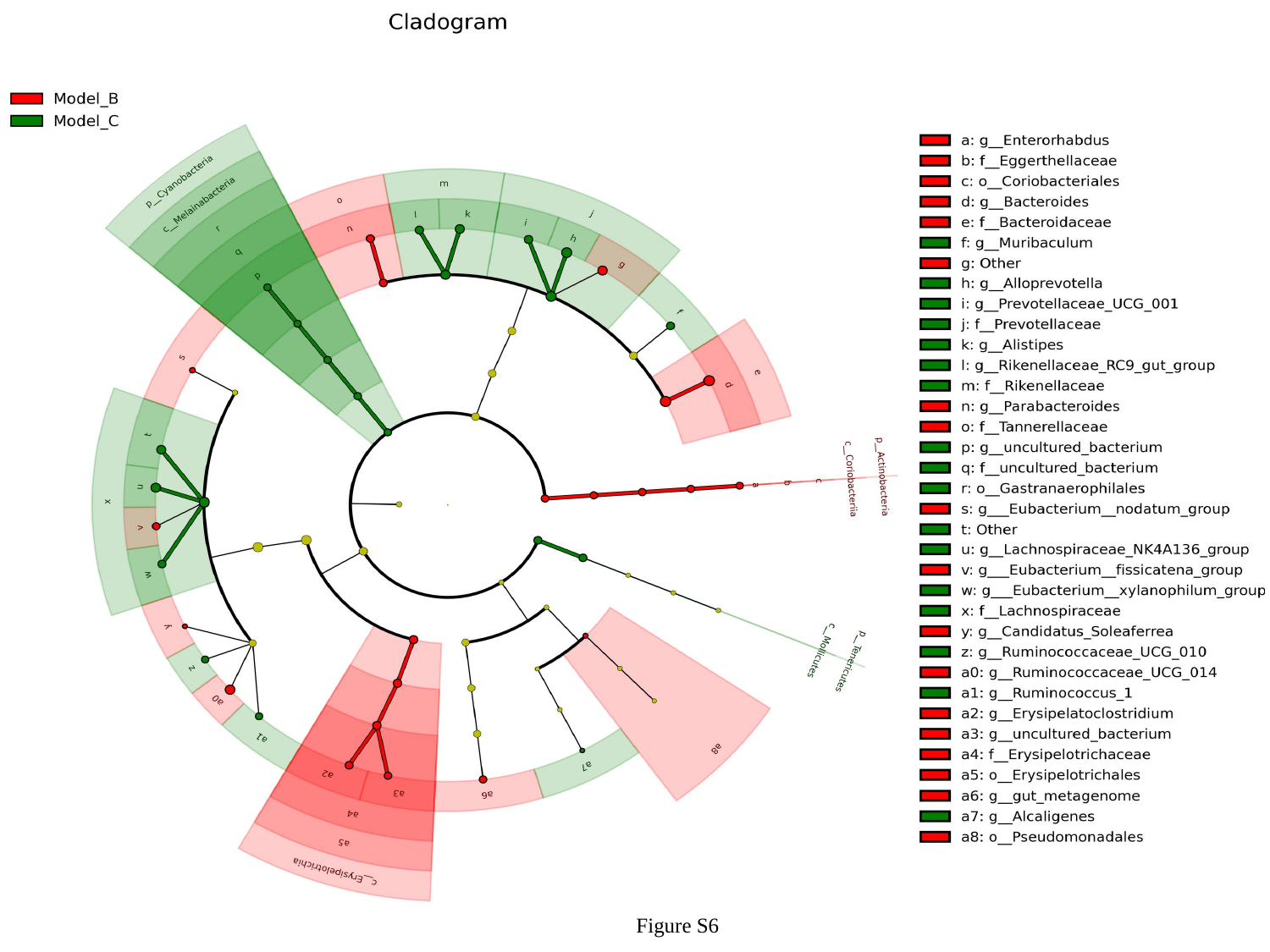

Figure S6

## Slide 7
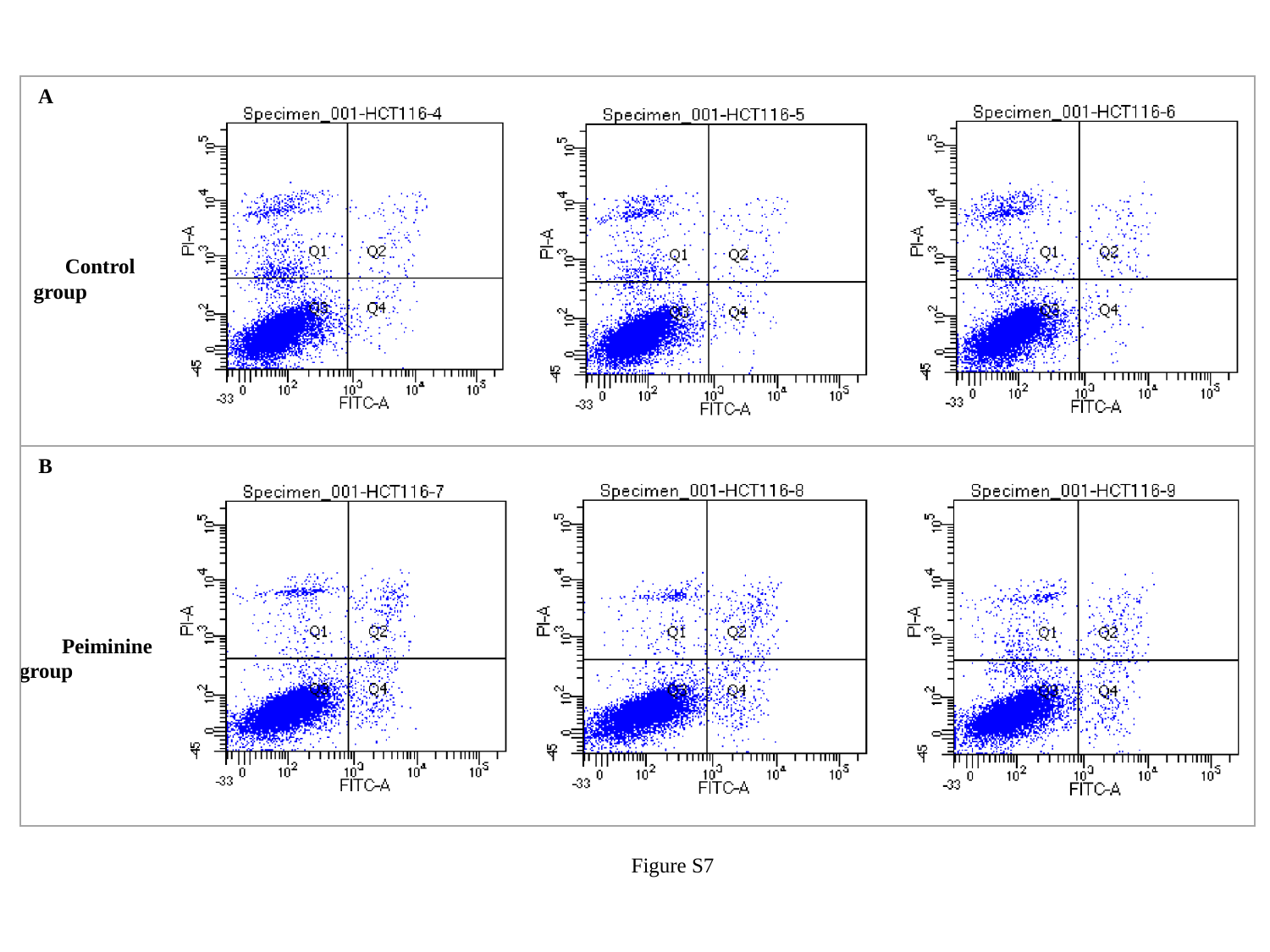

A
Control
group
Peiminine
group
B
Figure S7

## Slide 8
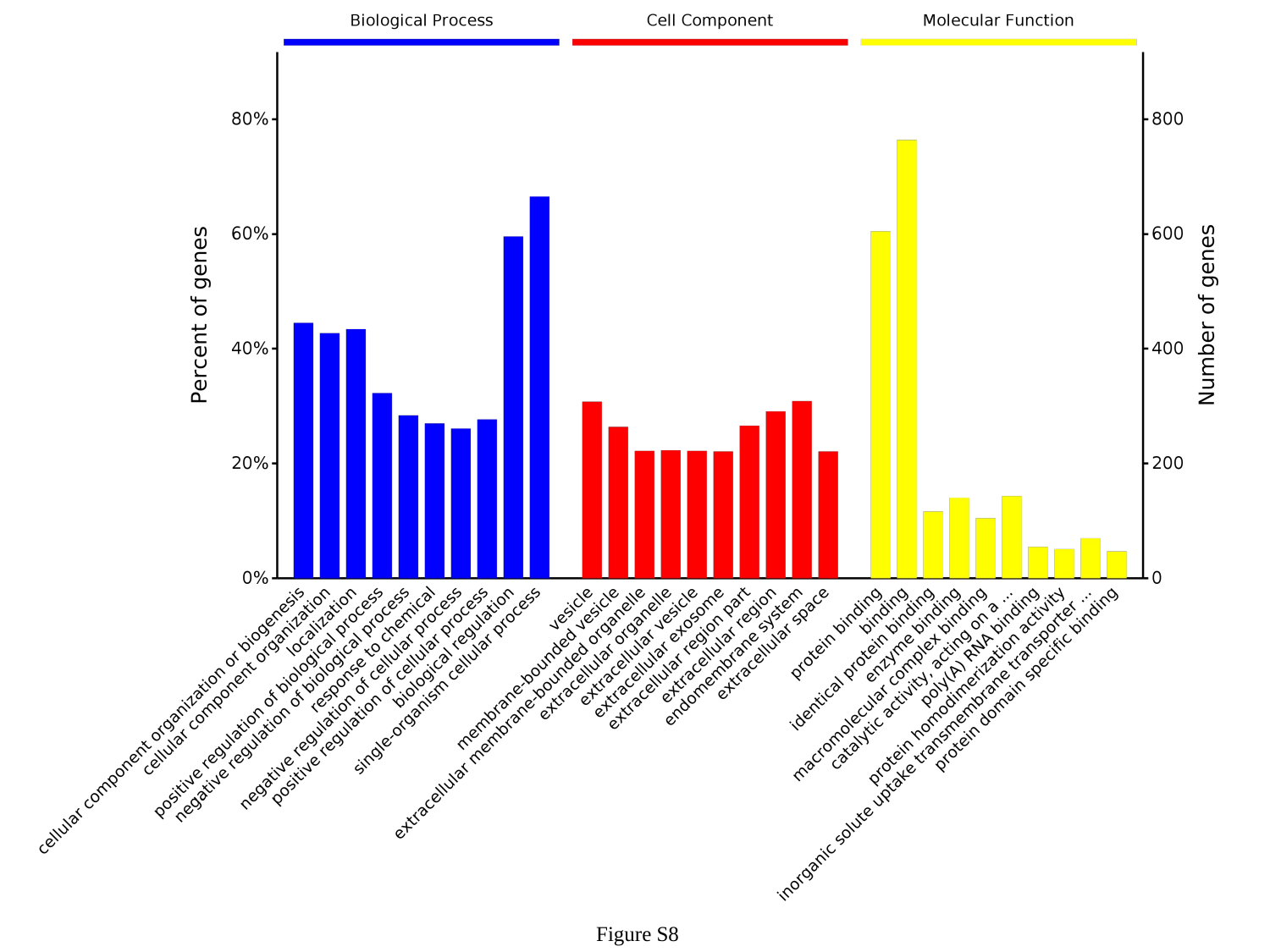

Figure S8
